# Magnetospinography visualizes electrophysiological activity in the cervical spinal cord

**DOI:** 10.1038/s41598-017-02406-8

**Published:** 2017-05-19

**Authors:** Satoshi Sumiya, Shigenori Kawabata, Yuko Hoshino, Yoshiaki Adachi, Kensuke Sekihara, Shoji Tomizawa, Masaki Tomori, Senichi Ishii, Kyohei Sakaki, Dai Ukegawa, Shuta Ushio, Taishi Watanabe, Atsushi Okawa

**Affiliations:** 1 Department of Orthopaedic Surgery, Tokyo Medical and Dental University, 1-5-45 Yushima, Bunkyo-ku Tokyo, 113-8510 Japan; 2 Department of Advanced Technology in Medicine, Graduate School of Tokyo Medical and Dental University, 1-5-45 Yushima, Bunkyo-ku Tokyo, 113-8510 Japan; 3 Applied Electronics Laboratory, Kanazawa Institute of Technology, Kanazawa-shi Ishikawa, 920-1331 Japan; 4 Ricoh Institute of Future Technology, RICOH COMPANY, LTD., 16-1 Shinei-cho, Tsuzuki-ku, Yokohama-shi Kanagawa, 224-0034 Japan

## Abstract

Diagnosis of nervous system disease is greatly aided by functional assessments and imaging techniques that localize neural activity abnormalities. Electrophysiological methods are helpful but often insufficient to locate neural lesions precisely. One proposed noninvasive alternative is magnetoneurography (MNG); we have developed MNG of the spinal cord (magnetospinography, MSG). Using a 120-channel superconducting quantum interference device biomagnetometer system in a magnetically shielded room, cervical spinal cord evoked magnetic fields (SCEFs) were recorded after stimulation of the lower thoracic cord in healthy subjects and a patient with cervical spondylotic myelopathy and after median nerve stimulation in healthy subjects. Electrophysiological activities in the spinal cord were reconstructed from SCEFs and visualized by a spatial filter, a recursive null-steering beamformer. Here, we show for the first time that MSG with high spatial and temporal resolution can be used to map electrophysiological activities in the cervical spinal cord and spinal nerve.

## Introduction

Imaging techniques are unquestionably important for the diagnosis of nervous system disease, but no single modality is sufficient to evaluate all aspects of nervous system function. Functional assessment with high temporal and spatial resolution is vital for diagnosing and treating spinal cord dysfunction. For example, cervical spondylotic myelopathy (CSM), in which the spinal canal narrows due to degenerative changes in the cervical spine, is one of the most common spinal cord disorders in the elderly^1–3^. Due to the mechanical and physiological pathology induced by compression of the spinal cord^4, 5^, patients with CSM show weakness and numbness in the upper extremities, gait deficit, spasticity of the lower extremities and bowel and bladder dysfunction^3^. However, some patients are clinically asymptomatic, even when spinal cord compression is apparent on imaging studies such as magnetic resonance imaging (MRI)^1, 6–12^. Additionally, the natural history of CSM varies and is unpredictable^13^, and the severity and features of the symptoms are diverse, even when similar degrees of spinal cord compression are seen on radiographic examination^5^. Therefore, for the early prediction of progression to myelopathy and the determination of treatment options, it is essential to evaluate spinal cord function and accurately identify the lesions responsible. For such applications, MR techniques such as functional MRI, diffusion tensor imaging and MR spectroscopy have been studied^14, 15^. Because the spinal cord is a narrow component compared with the brain, the resolution of these imaging techniques requires improvement.

Electrophysiological recording is another method for assessing spinal cord function. It has been used not only for intraoperative monitoring, but also for preoperative assessment to diagnose spinal lesions responsible for myelopathy and to predict progression to myelopathy in asymptomatic cases^16–21^. However, electrophysiological activity recorded at the body surface is not particularly suitable for recording conductive activity in deep and complex structures such as the spinal cord, spinal nerve and brachial plexus. Because surface potentials have relatively small amplitudes and are affected by the conductivity of tissues surrounding the nerve, the conduction velocity is not stable or accurate^22, 23^.

To record larger and clearer signals than surface potentials recorded from the body surface near the spine, spinal cord evoked potentials (SCEPs) have been used. SCEPs can be recorded using an epidural catheter electrode directly on the spinal cord in response to spinal or transcranial stimulation. These SCEPs are helpful for detecting responsive segments of the spinal cord in CSM^16, 24^ and have been mainly used by spinal surgeons. However, the insertion of an electrode into a narrowed cervical epidural space risks injury to the cervical spinal cord and requires considerable skill. Therefore, noninvasive yet precise methods for functional assessment of spinal cord activity are required. Magnetospinography (MSG) is one such possible method.

When a nerve is electrically stimulated, intra-axonal currents generate neuromagnetic fields according to Ampère’s circuital law (Supplementary Fig. S1). Although nine orders of magnitude smaller than the geomagnetic field^25^, these weak magnetic fields from biological structures can be detected in magnetically shielded rooms using sensitive magnetic sensors. Each sensor detects magnetic fields orthogonal to its sensor coil, and simultaneous recording from multiple sensors can visualize spatial expansion of the evoked magnetic field as a magnetic contour map. In addition, temporal and spatial expansion of the evoked action currents can be computationally reconstructed and visualized as a current map. Therefore, without recording electrodes, images of propagating action currents can be obtained.

Currently, magnetoencephalography (MEG) and magnetocardiography (MCG) are available for clinical use, but spinal imaging (MSG) has not progressed to clinical applicability, partly due to signal localization difficulties. The evoked magnetic fields are about one-tenth smaller than those of MEG and the evoked currents behave more intricately because of the structure of the spine. MEG instrumentation has helmet-shaped arrays of superconducting quantum interference device (SQUID) sensors and is most sensitive to cortical currents tangential to the skull^26^. Compared with MEG, MSG needs a higher sampling rate to record intra-axonal action currents propagating at higher velocity. Therefore, novel device and data analysis methods suitable for the spinal cord need to be developed.

Magnetoneurography (MNG), including MSG, has several advantages over electrophysiological recording for the assessment of nerve activity and dysfunction. First, MNG does not require large electrode arrays to scan a wide area. Conventional electrophysiological recording needs multiple recording points to reconstruct action currents. Second, action currents in nerves can be more accurately computationally reconstructed from evoked magnetic fields. As mentioned, evoked potentials recorded at the body surface are of greatly diminished amplitude and appear to propagate at different velocities than when recorded directly because they are easily distorted by the conductivity and shape of the volume conductor. Therefore, it is difficult to precisely localize sites of conduction block in deeply seated nerves such as the spinal cord, spinal nerve root, brachial plexus, and lumbar plexus by surface electrophysiological recording. In contrast, the magnetic field is not affected by electric conductivity, although it does attenuate with distance. Consequently, current sources, including intra-axonal action currents and volume currents, can be extrapolated from evoked magnetic fields. Importantly, action currents at any point in the scanned area can be reconstructed as if ‘virtual electrodes’ were in place, and the source reconstruction can visualize the pattern of action currents in the nervous system as a current map.

Recording of evoked magnetic fields in peripheral nerves has advanced in recent decades. In 1980, Wikswo and colleagues reported evoked magnetic fields from a frog sciatic nerve^27^. When the isolated nerve was inserted into a toroid pickup coil and stimulated, large magnetic fields (peak 60 pT) were measured; however, this method requires exposure and isolation of the nerve to achieve such large responses. Subsequent efforts focused on noninvasively recording nerve evoked magnetic fields at the body surface^28–36^. The development of magnetically shielded rooms and high-sensitivity SQUID sensors have made noninvasive recording possible by improving the signal-to-noise ratio. Additionally, noise reduction methods and source reconstruction techniques have improved.

In 1998, Mackert and colleagues^32^ reported magnetic fields around the lumbar spine in patients with lumbar disc hernia. The estimated conduction block positions were 2.5 cm caudal and 0.8–2.5 cm lateral to the site of nerve root compression. However, when reviewing the mechanism and early history of MNG, they pointed out that more sensitive measurement hardware and more accurate source reconstruction algorithms were required before MSG could be used in clinical practise.

Since 1999, our group has been working on MSG and MNG of peripheral nerves to noninvasively assess spinal cord and deep nerve dysfunction. To date, we have reported neuromagnetic fields in animals using MSG, propagation and conduction block of spinal cord evoked magnetic fields (SCEFs)^37–39^, magnetic fields evoked by synaptic activity in the lumbar spinal cord of the rabbit^40^, and the detailed characteristics of action currents and magnetic fields at the site of conduction block in isolated nerves^41, 42^. In addition, we have recorded neuromagnetic fields from the lumbar canal of human subjects^43^. In the process, we have made various improvements to our measurement system: a SQUID biomagnetometer^44^ with a better signal-to-noise ratio and lower consumption of liquid helium; use of an X-ray system that enables more precise localization; an optimal nerve stimulation method for larger signals; and artefact reduction and optimal signal processing for signal localization.

Noise reduction methods, including dual signal subspace projection (DSSP)^45^ and common-mode subspace projection (CSP)^46^, have allowed us to resolve magnetic fields that are small and/or buried in noise. In our early studies, we made assumptions about action currents and volume conductors for source localization^41, 42^ that proved useful for relatively simple systems, such as an isolated peripheral nerve, but were not satisfactory when applied to the spinal cord. In the present study, we used a spatial filter called a recursive null-steering (RENS) beamformer^47, 48^ that does not assume any volume conductor. The use of the RENS beamformer enabled us to more accurately reconstruct action currents in the spinal cord and the spinal nerve root. It should be emphasized that MSG can visualize the distribution of currents in the spinal cord. Conventional electrophysiological examinations rely on electric potential recording, because recording of action currents is difficult *in vivo*. Therefore, observation of action currents by MSG could help in neuroscientific research.

We report here for the first time that MSG can visualize propagating evoked currents in the cervical spinal cord of a CSM patient and healthy subjects, demonstrating that MSG has great potential for the noninvasive clinical assessment of the spinal cord.

## Results

### SCEFs and SCEPs in response to stimulation of the lower thoracic spinal cord

**Figure 1:**
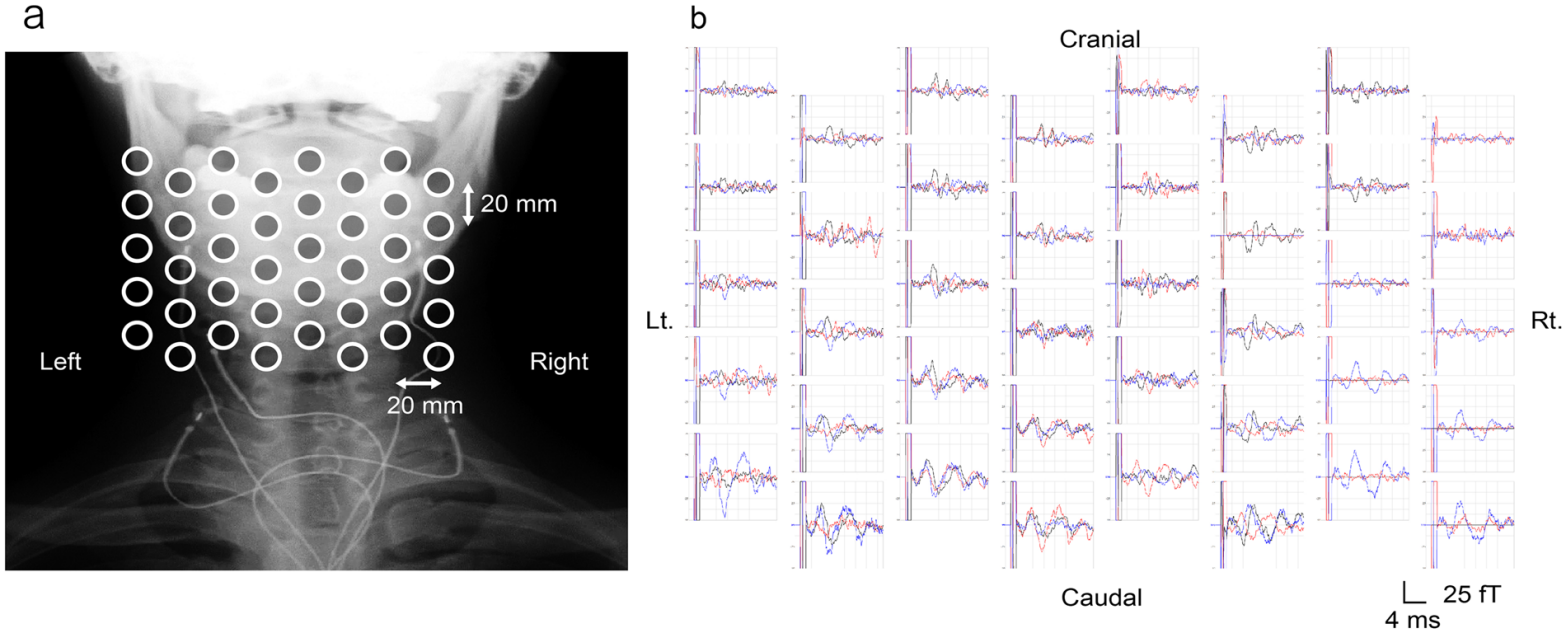
Spinal cord evoked magnetic fields (SCEFs) of a healthy subject measured after stimulation of the lower thoracic spinal cord. (**a**) Positions of the sensors superimposed on an X-ray image of a subject. (**b**) The three-directional magnetic fields recorded by each sensor. Black traces are magnetic fields in the ventral–dorsal direction relative to the cervical spinal cord (dorsal is upwards in the graphs). Red traces are magnetic fields in the left–right direction (right is upwards). Blue traces are magnetic fields parallel to the spinal cord (cranial is upwards). Some malfunctioning pickup coils show a flat line. The red signals, mainly generated from intra-axonal currents, are highest above the spinal cord. The polarity of the black and blue signals is reversed on each side of the spinal cord.

**Figure 2:**
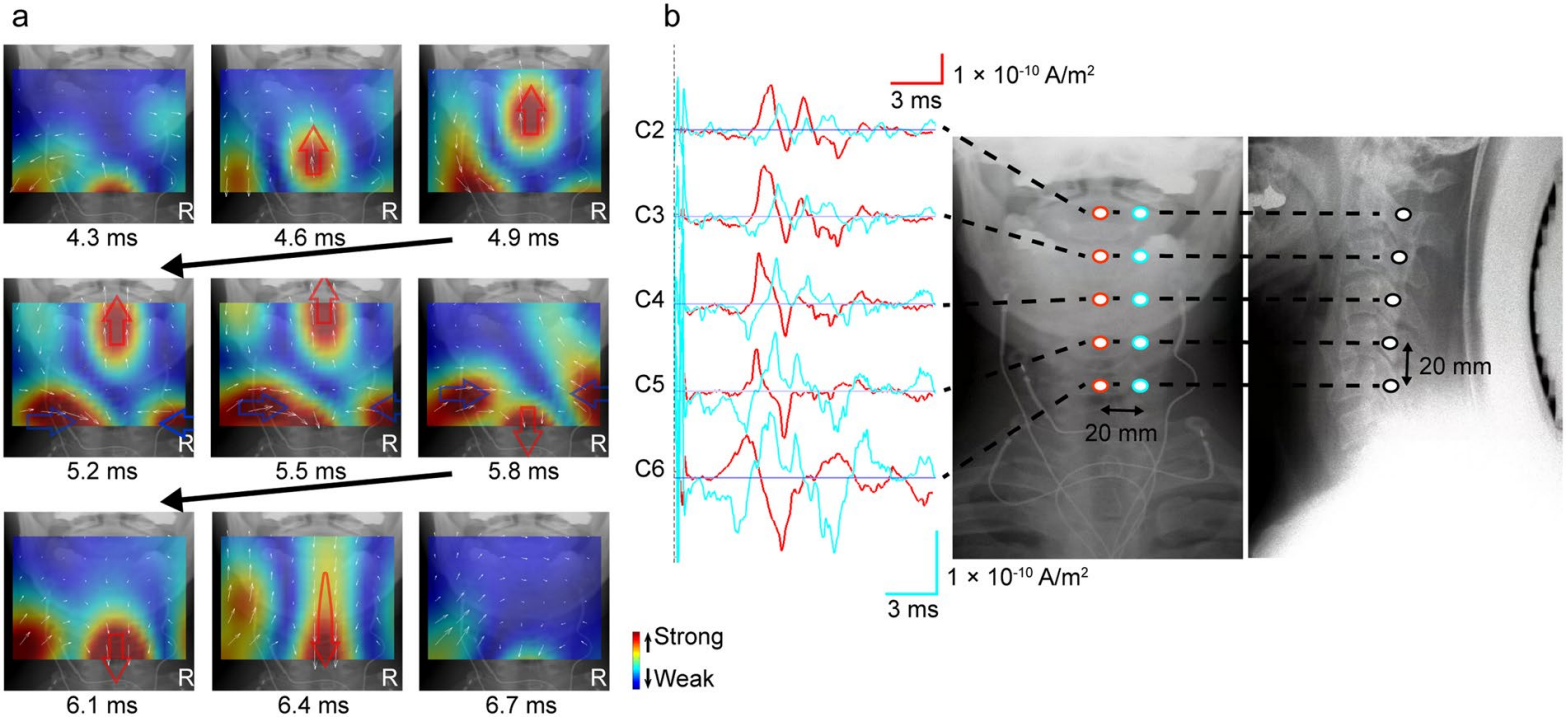
Reconstructed currents of a healthy subject measured after stimulation of the lower thoracic spinal cord. (**a**) Reconstructed current map. Currents at the level of the spinal canal were reconstructed by a recursive null-steering (RENS) beamformer and superimposed on an X-ray image. Current intensity is shown by a colour scale (red is higher). Small white arrows indicate current vectors. The leading component of the currents (large upwards-pointing red arrow) appeared 4.3 ms after stimulation and propagated cranially along the spinal canal. At 5.8 ms, the trailing component (downwards-pointing red arrow) appeared from the caudal side. Perpendicular currents (blue arrows) flowing towards the spine between the leading and the trailing components are also observed on both sides. (**b**) Waveforms of reconstructed currents at the “virtual electrodes”. Red traces are currents at the midline of the cervical spinal canal (the caudal to cranial direction is upwards). Blue traces are currents 20 mm lateral from the midline (upwards is towards the spinal canal).

Cervical SCEFs in response to stimulation of the lower thoracic cord were recorded from three healthy subjects. The magnetic fields had two to four phasic waveforms and propagated in the caudal to cranial direction (Fig. 1). The evoked currents reconstructed by the RENS beamformer and converted to a psuedocolour map are superimposed on an X-ray image of the cervical spine (Fig. 2a, Supplementary Video S2). Starting 4.3 ms after the stimulation, leading and trailing components of the intra-axonal currents appeared at the caudal-most recording site and propagated along the spinal canal (large red arrow). In addition, two other components appeared on either side of the spinal cord and flowed perpendicularly between the leading and trailing components (large blue arrows). These inflows also propagated cranially along with the intra-axonal currents. The waveforms of reconstructed currents at the midline of the cervical spinal canal (red) and 2-cm lateral (blue) are shown in Fig. 2b. The red traces are currents parallel (upwards is the caudal to cranial direction) and the blue traces indicate currents perpendicular (upwards is towards the spinal canal) to the spinal cord. The first wave in red is equivalent to the leading component in Fig. 2a (indicated by the upwards-pointing red arrow). The conduction velocity (CV) of the reconstructed currents was calculated from the peak of the first wave in red (mean of 64.3 m/s in the healthy subjects).

**Figure 3:**
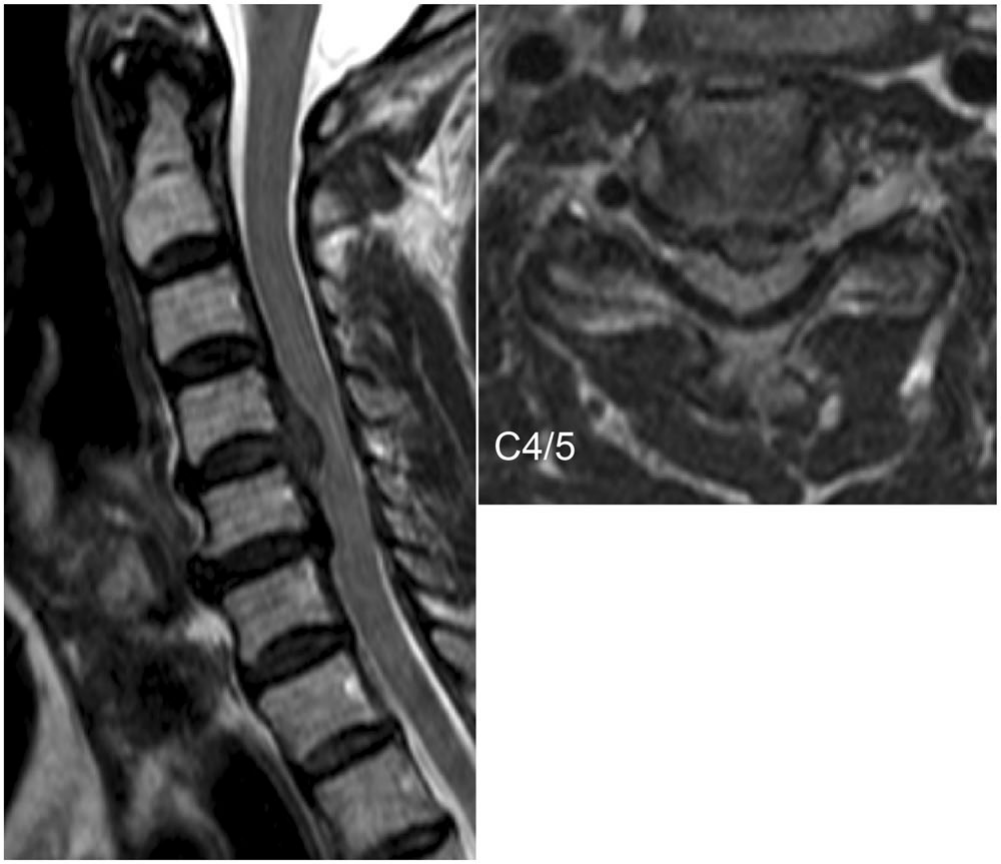
Preoperative MR image of a patient with cervical spondylotic myelopathy showing spinal stenosis. The patient was a 67-year-old male with C4/5 disc herniation and a small indentation of the C5/6 disc. The patient exhibited clumsiness of bilateral hands and gait disturbance.

**Figure 4:**
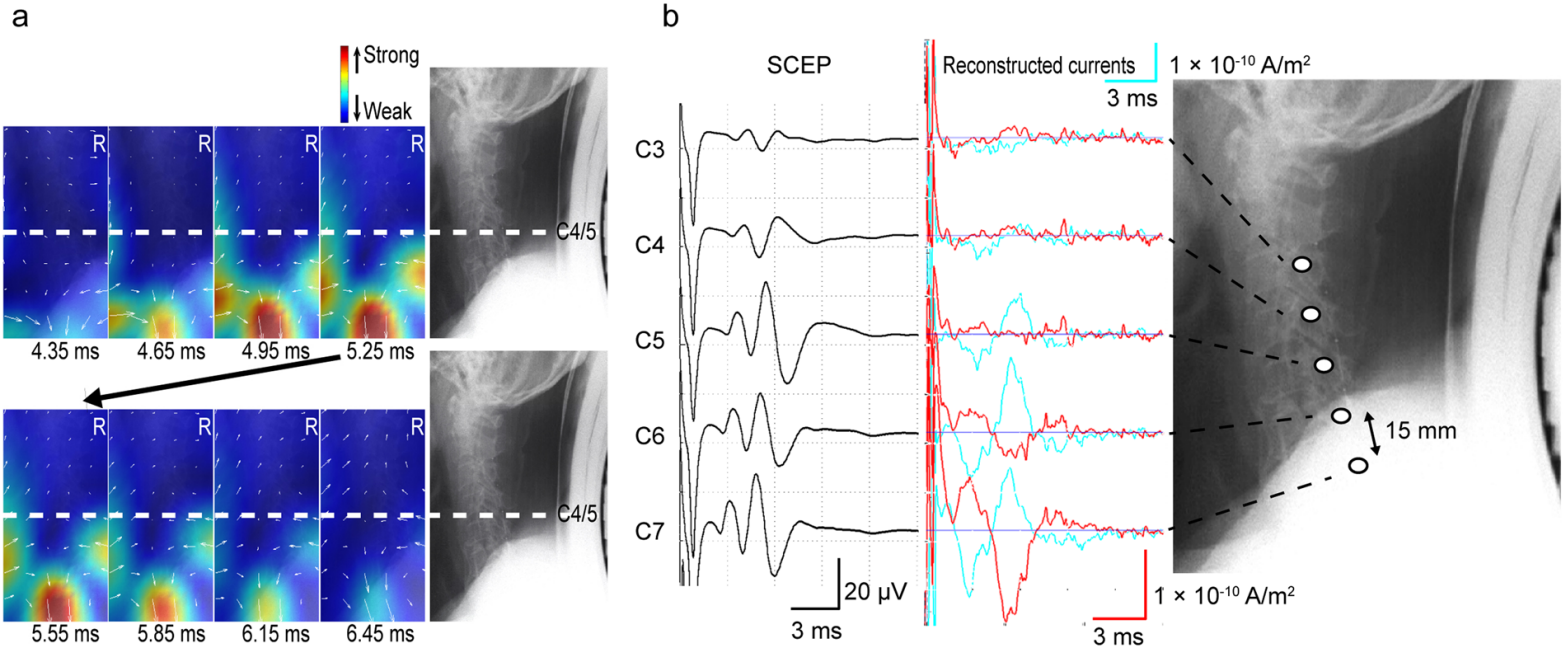
Reconstructed currents of the cervical spondylotic myelopathy patient. Currents are illustrated in a similar manner to Fig. 2. The current map is in the anterior–posterior direction, and the X-ray image of the lateral view shows the corresponding levels of the cervical spine. (**a**) In the reconstructed current map, the leading component is not illustrated because its intensity is much lower than that of other currents. When the perpendicular components stopped and disappeared caudal to C4/5, the trailing component simultaneously disappeared caudal to C5/6. (**b**) The left graphs are the ascending spinal cord evoked potentials (SCEPs) by stimulation of the lower thoracic cord showing conduction block at the C4/5 disc level. The right graphs are the reconstructed currents at the midline of the cervical spinal canal (red) and 2 cm lateral (blue). The leading component (the first waveform in red) attenuated and disappeared through C4–6, and the trailing component (the second waveform in red) disappeared at C5/6. The perpendicular inflow components greatly attenuated at C4/5 (the second waveform in blue).

MRI of the CSM patient revealed substantial spinal cord compression at the C4/5 disc level and milder compression at the C5/6 disc level (Fig. 3). The ascending SCEPs in response to stimulation of the lower thoracic cord showed propagation block at the C4/5 disc level (Fig. 4b, left). The reconstructed current map (Fig. 4a, Supplementary Video S3) revealed current distribution patterns caudal to C4/5 similar to those recorded in healthy subjects. However, the leading component is not illustrated because it was much smaller than that of other currents. When the perpendicular components stopped and disappeared before C4/5, the trailing component simultaneously disappeared before C5/6. The red traces in Fig. 4b show that the leading component (the first wave) attenuated as it passed through C4–6 and the trailing component (the second wave) disappeared at C5/6. The perpendicular inflow components were also largely attenuated at C4/5 (the second wave in blue, Fig. 4b right). The latency and waveform changes of the reconstructed currents corresponded to those of the SCEPs (Fig. 4b), with very similar CVs below C6 (53.3 m/s for the reconstructed currents and 55.6 m/s for the SCEPs).

### Cervical SCEFs in response to median nerve stimulation at the elbow

**Figure 5:**
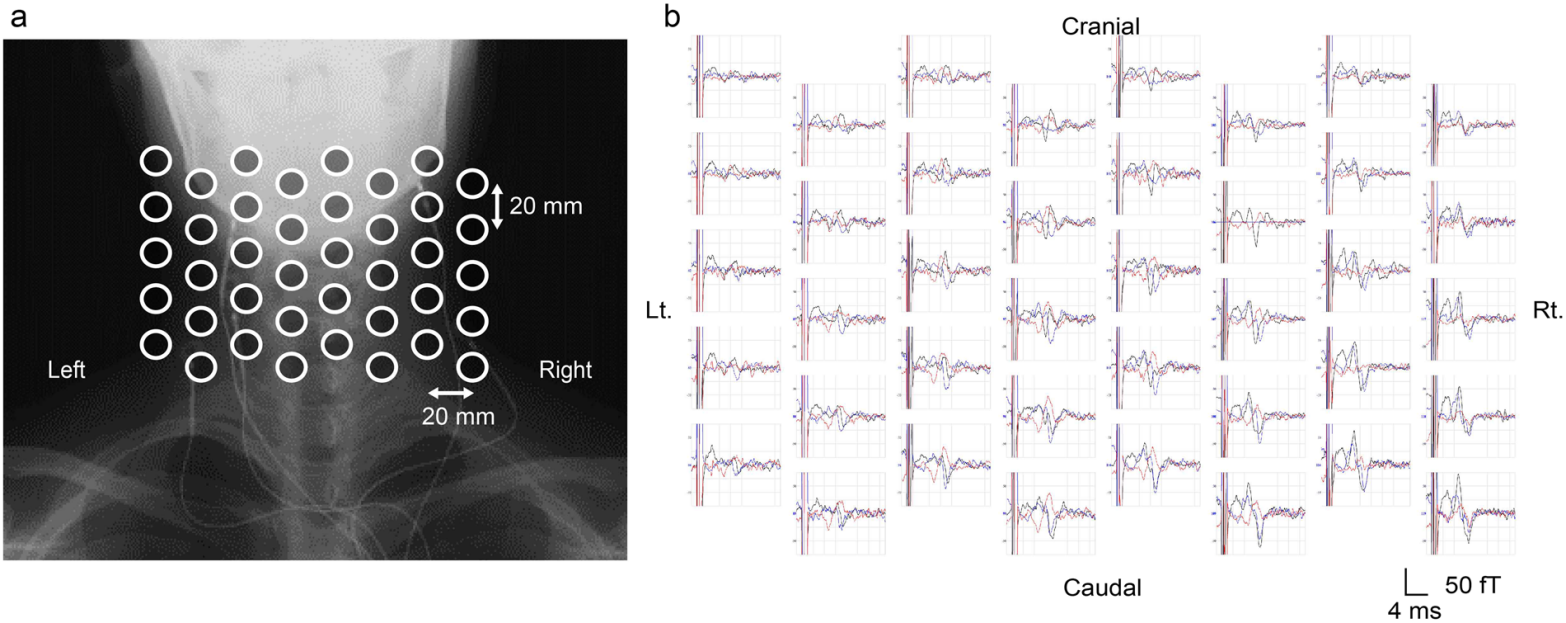
Spinal cord evoked magnetic fields (SCEFs) in response to stimulation of the median nerve at the elbow. (**a**) Positions of the sensors. (**b**) SCEFs illustrated in a manner similar to Fig. 1. SCEFs were mainly recorded from the right side of the spinal cord.

Cervical SCEFs were recorded in response to stimulation of the median nerve in ten subjects (20 nerves). The first peak appeared at about 5.5 ms post-stimulus and the magnetic fields consisted of two to four distinct phases (Fig. 5). The reconstructed evoked currents appeared more dispersed than after stimulation of the lower thoracic cord.

**Figure 6:**
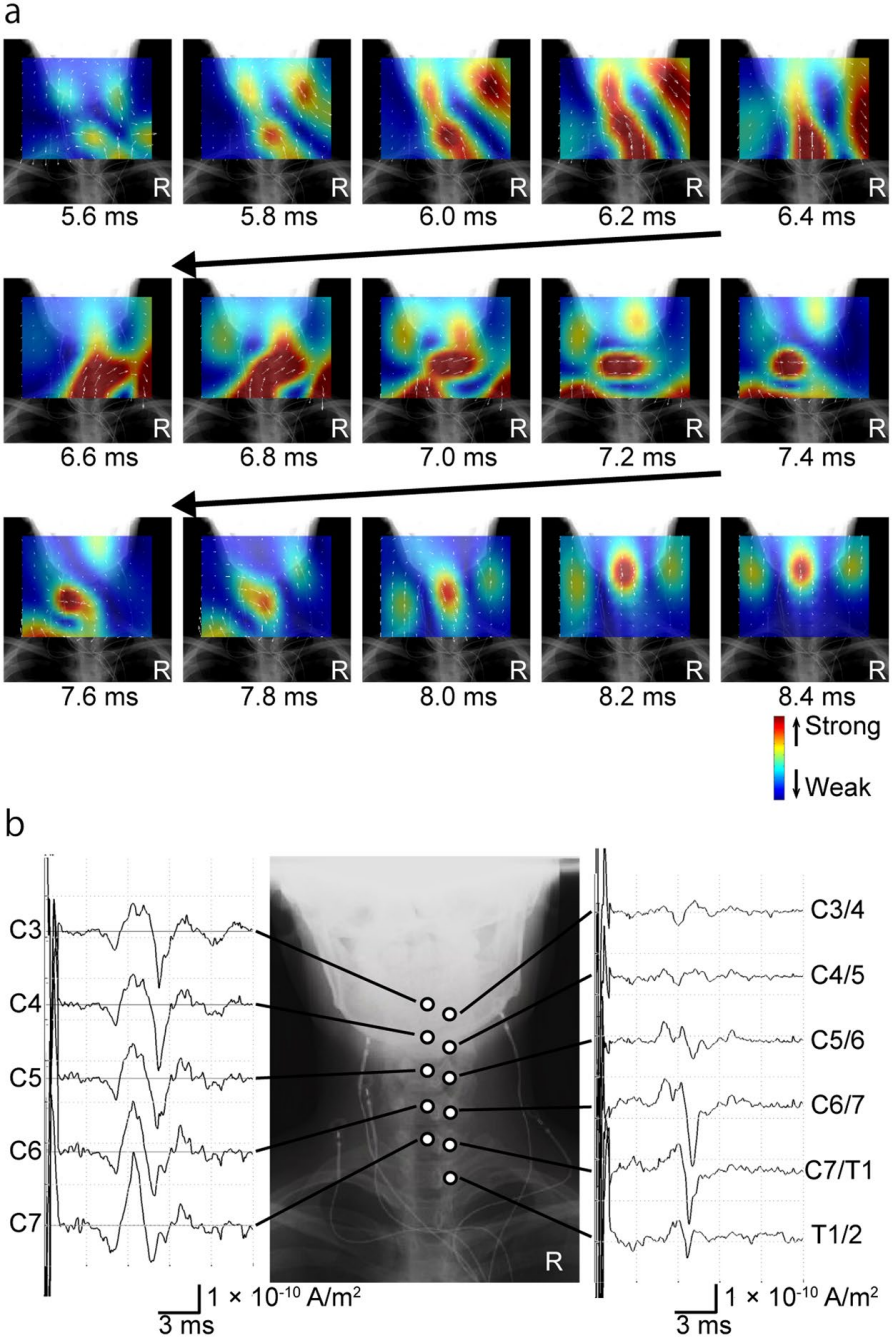
Reconstructed currents after stimulation of the right median nerve at the elbow. (**a**) Reconstructed current map. The currents flowed into the spinal canal from the right side and propagated cranially 5.6–6.4 ms post-stimulus. Rotated currents were observed 6.6–7.6 ms post-stimulus. Trailing currents propagated cranially 7.8–8.4 ms post-stimulus. (**b**) Reconstructed currents at the midline of the cervical spinal canal (C3 to C7) and at the adjacent intervertebral foramina (C3/4 to Th1/2). The first peak of the reconstructed currents at the midline of the cervical spinal canal did not propagate, but subsequent currents did (left graphs). Currents flowing into the intervertebral foramina were larger at C5/6 to C7/Th1 (right graphs).

In a representative case, the currents flowed into the spinal canal from the stimulated side and propagated cranially (5.6–6.4 ms). Subsequently, rotated currents were observed (6.6–7.6 ms) and trailing currents propagated cranially (7.8–8.4 ms) (Fig. 6a, Supplementary Video S4). The first peak of the reconstructed currents at the midline of the cervical spinal canal did not propagate, but subsequent currents did (left graph in Fig. 6b). The reconstructed currents at adjacent points of the intervertebral foramina (C3/4 to Th1/2) are shown in the right graph in Fig. 6b. The currents flowing into the intervertebral foramina were larger at C5/6 to C7/Th1.

**Figure 7:**
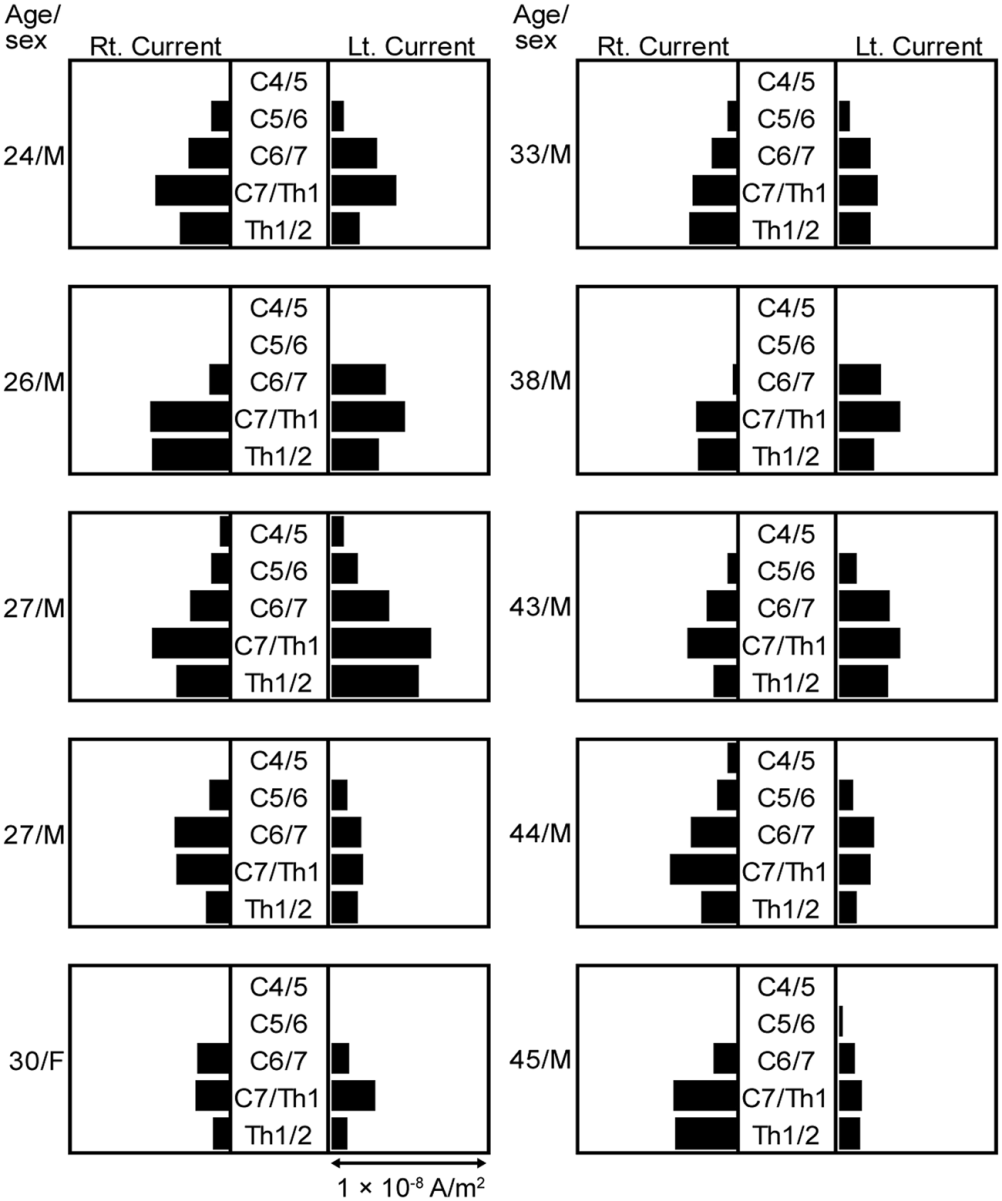
Individual variations in the peak currents flowing into the intervertebral foramina. In most subjects, the largest currents flowed through C6/7 or C7/Th1. Currents passing through the C4/5 intervertebral foramen (outlet of root C5) were observed in two of the ten subjects, a 27-year-old man (upper one) and a 44-year-old man.

The CVs calculated from the peak latency at the C5–6 level ranged from 53.3 to 120 m/s (*n* = 20; mean, 73.0 ± 15.8 m/s). Individual variations in peak currents flowing into the intervertebral foramina are shown graphically in Fig. 7. The magnitude of current inflow differed among the subjects, but the maximal currents flowed through C6/7 or C7/Th1 in most subjects.

## Discussion

Noninvasive surface MSG visualized propagating spinal excitation in response to thoracic spinal cord and median nerve stimulation with high spatial and temporal resolution. The signal propagation pattern and velocity were consistent with known innervation pathways and electrophysiological properties, respectively. Most significantly, MSG allowed for the visualization of conduction block at the site of spinal stenosis in a CSM patient. These results demonstrate that MSG is a useful method for the diagnosis and treatment evaluation of peripheral nerve and spinal cord diseases. Given that conventional electrophysiological examinations depend on the recording of electric potentials, observation of action currents reconstructed by MSG could expand the possibilities for neuroscientific research.

In the first series of experiments, cervical SCEFs propagating along the spinal canal were measured in healthy subjects. The reconstructed currents contained leading and trailing components that represented intra-axonal currents in the spinal cord, and two perpendicular inflowing components that represented volume currents flowing towards the centre of depolarization (Fig. 2a, Supplementary Video S2).

For the CSM patient, the reconstructed current map showed that the leading component was blocked around C4/5 and, when the perpendicular inflow currents became attenuated at C4/5, the trailing component simultaneously disappeared at C5/6 (Fig. 4a, Supplementary Video S3). Because the perpendicular inflow currents correspond to volume currents flowing into depolarization sites, attenuation of the perpendicular currents at C4/5 indicates conduction block around C4/5. As we previously reported, the trailing component also disappeared when the perpendicular inflow currents disappeared^41, 42^, because it too was generated by the depolarization site.

In the second series of experiments, propagating action currents in the spinal canal were reconstructed from cervical SCEFs in response to stimulation of the median nerve. As shown in Fig. 6b, the first wave at the midline of the cervical spinal canal did not propagate cranially or caudally. Considering its latency, this wave appeared to be derived from conductivity changes as the depolarization propagated along the spinal nerve through the intervertebral foramen to the spinal cord in the spinal canal and cerebrospinal fluid. Further research, including simultaneous recording of SCEFs and SCEPs in a larger number of subjects, is needed to elucidate the mechanism involved. However, the subsequent waves did propagate along the spinal cord. From 6.6 to 7.6 ms post-stimulus, the reconstructed currents appeared to rotate in the spinal canal (Fig. 6a, Supplementary Video S4). In our previous studies, similar rotating magnetic fields were recorded around the intervertebral foramina^40, 43^. These rotating currents may reflect the summation of synaptic activity and currents from the dorsal column pathway. However, considering the complexity of the entry zone of the spinal nerve root into the spinal cord^49–51^, further investigation is needed.

MSG also allowed for visualization of propagating activity through the intervertebral foramina and into the spinal cord following surface median nerve stimulation. Anatomically, the median nerve is usually innervated from C6–Th1 but less often by C5^52^. Consistent with the typical innervation pattern, the currents were largest at the C6/7 intervertebral foramen (outlet of root C7) or the C7/Th1 intervertebral foramen (outlet of root C8) in most subjects while currents passing through the C4/5 intervertebral foramen (outlet of root C5) were observed in two of the ten subjects, a 27-year-old man and a 44-year-old man (Fig. 7).

In pioneering work, Curio and colleagues^29^ recorded magnetic fields around the brachial plexus and neck using a single SQUID sensor. In particular, as described in a review by Mackert^53^, the conduction block positions were localized down to the centimetre scale in patients with lumbar disc herniation. While these studies contributed to the development of MNG, greater accuracy was thought necessary for clinical application. Limited accuracy in these early reports stemmed from mechanical restriction of the SQUID sensor and inadequate source reconstruction techniques. Specifically, previous reconstruction techniques using dipole estimation and assuming some volume conductor reduced the accuracy of current source localization. Although we also previously recorded SCEFs from humans, the combined efforts of a multidisciplinary team were needed for clinical application. In summary, the following advances have been made: a SQUID biomagnetometer suitable for the cervical spine with a better signal-to-noise ratio and lower consumption of liquid helium; an X-ray system for better localization; a nerve stimulation method that enables a larger response and the ability to subsequently minimize artefacts; and signal analysis techniques optimal for MSG. In this study, we used a more advanced reconstruction technique that does not restrict the number or direction of currents^47, 48^, as well as the latest SQUID sensor with design improvements for MSG^44^. This report demonstrates the feasibility of MSG for high temporal and spatial resolution imaging of spinal activity and for the localization of conduction block.

However, there are some limitations to this study. Further research should involve a greater number of subjects to enable precise localization of the conductive block in the spinal cord and to elucidate detailed electrophysiological activities at the entry point of the spinal nerve root into the spinal cord. Additionally, the instrumentation of MSG is still experimental and there is a need to stimulate global research to accumulate knowledge about SCEFs.

In summary, we have shown for the first time that MSG can visualize with high spatial and temporal resolution propagating spinal excitation in response to stimulation of the thoracic spinal cord and median nerve. MSG may be a clinically useful modality not only for the detection of spinal tract dysfunctions such as CSM, but also for the detection of spinal root and dorsal horn dysfunction, considering recent electrophysiological studies of radiculopathy and brachial plexus injury^54, 55^. A combination of MSG and peripheral nerve stimulation could be used in the noninvasive assessment of spinal cord function for basic research and clinical diagnosis.

## Methods

Two series of MSG recordings were performed. In the first series, the lower thoracic spinal cord was electrically stimulated and the magnetic fields of the cervical spinal cord were recorded. In the second series, magnetic fields of the cervical spinal cord were recorded in response to stimulation of the median nerve at the elbow.

### MSG system

**Figure 8:**
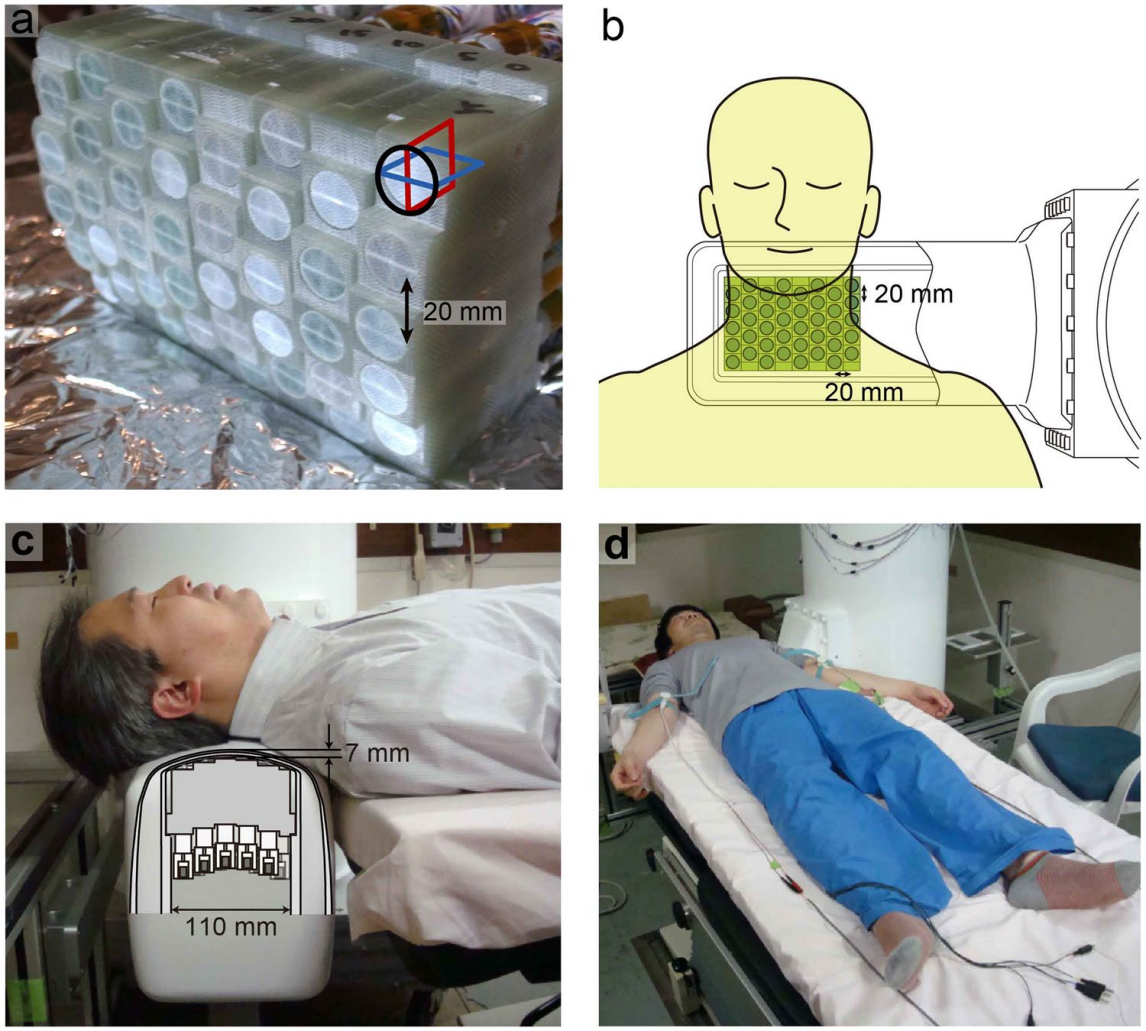
The 120-channel superconducting quantum interference device (SQUID) biomagnetometer system used for the magnetospinography. (**a**) Individual SQUID sensors are arranged in an eight-by-five configuration at 20-mm intervals. Each sensor has three perpendicular pickup coils to detect magnetic fields in three orthogonal directions. (**b**) Overhead view of the sensor and subject as shown in Figs 1 and 5. (**c**) Cross-sectional image of the cryostat protrusion. The central grey area indicates the sensor array shown in (**a**). The sensors are arranged in the vertical direction and positioned in close contact with the upper inwall of the protrusion. The subject was in the supine position on a table with the posterior neck on the protrusion holding the SQUID sensor array inside. The upper surface of the protrusion is curved to fit the lordosis of the cervical spine. (**d**) Both median nerves were alternatively stimulated at the anterior of the elbow joint. Volar splints to suppress evoked movements are not shown here.

All recordings were performed in a magnetically shielded room using a 120-channel SQUID biomagnetometer system developed by the Applied Electronics Laboratory, Kanazawa Institute of Technology^44^ (Fig. 8). The device has two main distinguishing features: an array of vector-type SQUID sensors and a uniquely designed cryostat especially optimized for recording SCEFs in the cervical and lumbar regions.

The sensor array is composed of 40 SQUID magnetic flux sensors arranged in an eight-by-five matrix-like configuration at 20-mm intervals (Fig. 8a). Each sensor is a vector-type gradiometer^56^, which has three pickup coils oriented orthogonally to one another to simultaneously detect three-dimensional magnetic fields. The baseline length of the gradiometric pickup coils is extended to 68 mm considering the deep magnetic sources in the spinal cord. The typical noise level of the SQUID sensors was less than 4 fT/Hz^1/2^.

The cryostat, used to maintain the superconducting state of the SQUID sensors, is a cylindrical plastic vessel holding liquid helium, characterized by a horizontal protrusion from its side surface. The sensor array is arranged in the protrusion in the upwards direction along its upper inwall. Subjects in the supine position put their neck on the protrusion, which is mildly curved to fit the posterior surface of the neck (Fig. 8b,c). The cool-to-warm separation at the upper surface of the protrusion is less than 7 mm, whereas that of the conventional MEG system is around 20 mm. This structure of the cryostat allows the SQUID sensors to be closer to the magnetic sources in the spinal cord and obtain a better signal-to-noise ratio.

### Signal processing

The position and intensity of the current sources were reconstructed from the evoked magnetic fields, and the current waveforms in the spinal canal and the intervertebral foramen were retrieved as if they were recorded using virtual recording electrodes. For positional information, magnetic signals from marker coils on the surface of the subject’s neck were recorded, and X-ray images of the subject and the SQUID sensor were obtained.

The measured data of SCEFs were first processed to remove stimulus-induced artefacts. For this purpose, the dual signal subspace projection (DSSP) algorithm^45^ was applied to obtain the artefact-removed data. The DSSP algorithm first projects the columns of the measured data matrix onto the inside and outside of the spatial-domain signal subspace, creating a set of two preprocessed data matrices. The intersection of the row spans of these two matrices is estimated as the time-domain interference subspace. The original data matrix is projected onto the subspace that is orthogonal to this interference subspace to remove the interference. The DSSP algorithm therefore does not require separate artefact measurements. The algorithm was detailed in a previous report^45^.

To record cervical SCEFs after stimulation of the median nerve at the elbow, we used another noise reduction method named common-mode subspace projection (CSP)^46^. This algorithm requires separate artefact measurements and estimates the intersection of row spans between the signal and artefact data matrices as the interference subspace. The CSP algorithm is effective even when artefact sources are located in the vicinity of the signal source space, although the total measurement time for the CSP algorithm is twice that of the DSSP algorithm. We used the CSP algorithm in our earlier experiments and switched to the DSSP algorithm in later ones to reduce the recording time.

The source analysis was then applied to the artefact-removed data. In the source analysis, using the obtained X-ray images of the subject and the SQUID sensor, the location of the spinal cord with respect to the sensors is estimated and a curved plane containing the spinal cord is determined as the reconstructed region. The spinal cord source activity is then reconstructed on this plane. Here, a spatial filter algorithm, the RENS beamformer^47, 48^, was applied. The RENS beamformer is derived based on the beam response optimization, and its weight is derived by a recursive procedure. The advantage of the RENS beamformer is its applicability to a single-time sample data. Its spatial resolution is much higher than that of the existing source imaging algorithms that may be applied to single-time samples, such as the minimum-norm method. In practice, the dynamics of the source activity was reconstructed in a time-point-by-time-point manner by applying the RENS beamformer to single-time sample data. The resultant source image was overlaid onto the patient’s X-ray image to obtain a movie showing the reconstructed electrophysiological activity of the spinal cord.

All procedures in this study were approved by the Ethics Committee of Tokyo Medical and Dental University and were performed in accordance with the Declaration of Helsinki. We obtained written informed consent and releases for images and photographs from all participants.

### Cervical SCEF and SCEP in response to lower thoracic spinal cord stimulation

The subjects were three healthy volunteers (36, 41 and 45 years of age; 178, 174 and 174 cm in height) without nervous system disease and one 67-year-old male patient with CSM scheduled for surgery. Before recording, a catheter electrode for spinal cord stimulation was transcutaneously inserted into the epidural space at the 11th thoracic spine level. The subject was in the supine position on a table in a magnetically shielded room with the posterior neck on the SQUID sensor (Fig. 8c). The thoracic spinal cord was electrically stimulated by the epidural electrode (17 Hz; monophasic square waves; 0.3-ms width; 3.5–6.6 mA constant current) and the evoked magnetic fields were recorded from 40 sensors at the surface of the posterior neck. Evoked responses were acquired at a sampling rate of 40 kHz with 100 Hz to 5 kHz analogue bandpass filtering. The averaged records of 4,000 individual responses were analysed. DSSP^45^ was then applied to reduce artefacts from the electrical stimulation and the electric devices.

The evoked currents were reconstructed by the RENS beamformer^47, 48^ and superimposed on the X-ray image to visualize their distribution, intensity and velocity. The current waveforms were retrieved from the virtual recording electrodes at each vertebral level at the midline of the cervical spinal canal and the 2-cm lateral line.

Another catheter electrode (five-polar; 15-mm intervals; Unique Medical Japan) was inserted transcutaneously into the cervical epidural space of the patient with CSM to record preoperative SCEPs. The lower thoracic spinal cord was electrically stimulated with an epidural electrode at the 11th thoracic spine level, as described above. The SCEPs at five points on the cervical spinal cord were recorded with reference to the earlobe and compared to the evoked magnetic fields and the reconstructed action currents.

### Cervical SCEFs after stimulation of the median nerve at the elbow

The subjects were ten healthy right-handed volunteers, 24–45 years of age (mean, 33.7 years), 156–175 cm in height (mean, 167.8 cm). In our earlier studies, we found that a larger neural signal is necessary to evaluate SCEFs and reconstruct currents at this time. Therefore, we stimulated the median nerve at a more proximal level than usual. The median nerve was stimulated at the anterior of the elbow joint (3 Hz; duration, 0.3 ms) at 1.5 times the motor threshold (intensity, 4.6–9.0 mA). The arm to be stimulated was fixed with a volar splint to suppress evoked movements. The right and left nerves were alternately stimulated and evoked magnetic fields were recorded from 40 sensors at the surface of the posterior neck (Fig. 8d). Two-thousand recordings of evoked magnetic fields were averaged from both sides. To measure magnetic fields from the stimulation artefact, the skin at the elbow 2–3 cm from the median nerve was electrically stimulated and 1,500 recordings were averaged. CSP^46^ was applied to the averaged data for artefact removal. The evoked currents were reconstructed by the RENS beamformer^47, 48^ using the data after artefact reduction and visualized as in the other recording conditions.

### Data availability

The data that support the findings of this study are available from the corresponding author upon reasonable request.

## Electronic supplementary material

Supplemtary Information

Supplementary video S2

Supplementary video S3

Supplementary video S4
