# Supplementary material for: Magnetospinography visualizes electrophysiological activity in the cervical spinal cord
Source: Sci Rep. 2017 May 19;7:2192. doi: 10.1038/s41598-017-02406-8 (PMC5438392; doi:10.1038/s41598-017-02406-8)

**Supplementary Information:**

# Magnetospinography visualizes electrophysiological activity in the cervical spinal cord

Satoshi Sumiya, MD[1]; Shigenori Kawabata, PhD[*,1,2]; Yuko Hoshino, PhD[2]; Yoshiaki Adachi, PhD[3]; Kensuke Sekihara, PhD[2]; Shoji Tomizawa, PhD[1]; Masaki Tomori, PhD[1]; Senichi Ishii, PhD[1]; Kyohei Sakaki, PhD[1]; Dai Ukegawa, PhD[1]; Shuta Ushio, MD[1]; Taishi Watanabe[4]; Atsushi Okawa, PhD[1]

**Supplementary Figure S1.** Schematic illustration of intra-axonal currents (red arrows), volume currents (yellow arrows flowing outside the nerve) and magnetic fields (green arrows) resulting from intra-axonal currents (magnetic fields generated by volume currents are not illustrated here).

**Supplementary Video S2.** Temporal changes in reconstructed currents of a healthy subject measured after stimulation of the lower thoracic spinal cord. A movie corresponding to Figure 2a.

**Supplementary Video S3.** Reconstructed currents of the cervical spondylotic myelopathy patient. A movie corresponding to Figure 4a.

**Supplementary Video S4.** Reconstructed currents after stimulation of the right median nerve at the elbow. A movie corresponding to Figure 6a.

# Supplementary figure 1

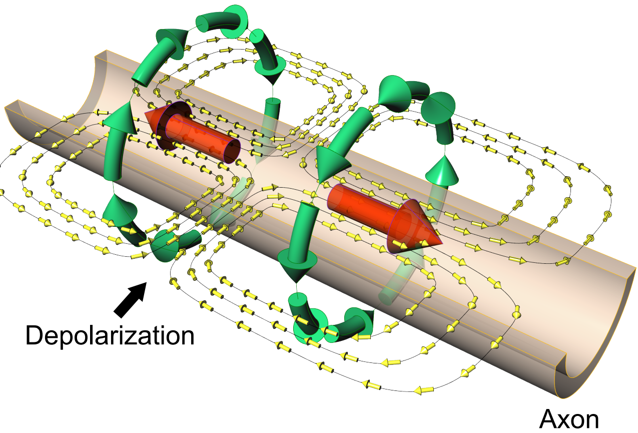

Supplement: Supplementary file 1 — Supplemtary Information [file 41598_2017_2406_MOESM1_ESM.pdf]
